# A more rapid method for transformation of *Helicobacter pylori*

**DOI:** 10.1128/msphere.00005-25

**Published:** 2025-01-31

**Authors:** Caroline D. Skene, Richard L. Ferrero

**Affiliations:** 1 Centre for Innate Immunity and Infectious Diseases, Hudson Institute of Medical Research 518514 , Clayton, Victoria, Australia; 2 Department of Molecular and Translational Sciences, Monash University, Clayton, Victoria, Australia; 3 Department of Microbiology, Biomedicine Discovery Institute, Monash University, Clayton, Victoria, Australia; University of Michigan-Ann Arbor, Ann Arbor, Michigan, USA

**Keywords:** *Helicobacter pylori*, naturally competent, transformation, mutagenesis, pathogen

## Abstract

*Helicobacter pylori* is a major causative agent in several upper gastroduodenal tract diseases, including gastric cancer. The development of methods to genetically manipulate *H. pylori* by natural transformation has allowed a greater understanding of its biology and role in these diseases. Nevertheless, the transformation methods used for *H. pylori* are time-consuming, requiring growth of these fastidious and slow-growing bacteria from −80°C stocks. The aim of the study was to develop a more rapid and convenient method for generating *H. pylori* mutants. We describe here a method in which competent *H. pylori* bacteria can be stored at −80°C and used in transformations on the day of resuscitation, similar to methods routinely used for *Escherichia coli*. This means that transformation can be performed at will and that transformants can be obtained within days, rather than weeks. Furthermore, we show that bacteria remain competent for at least six months storage at −80°C and that the method is applicable to strains with varying levels of natural competence. Transformation efficiencies of the bacteria varied between 10^1^ and 10^6^ transformants/total colony-forming units/µg donor DNA, depending on the strain. We suggest that this improved method will facilitate studies on *H. pylori* and, moreover, may be applicable to other naturally transformable pathogens with fastidious growth requirements and requiring ultra-low temperature refrigeration for long-term preservation.

**IMPORTANCE:**

Genetic manipulation is an important tool in the study of pathogenic bacteria and their interactions with the host. Many pathogenic bacteria are naturally transformable; however, transformation experiments can be impeded by the slow-growing and fastidious nature of some species. One such bacterium is *Helicobacter pylori*, which requires resuscitation from −80°C and multiple subcultures prior to transformation. The method described in the current study uses a simple modification of a conventional method of natural transformation. Using this method, competent *H. pylori* bacteria can be stored for long periods (at least six months) and resuscitated as needed for use in experiments. The method circumvents the need for multiple and lengthy subcultures prior to transformation, nor does it involve costly materials, complicated procedures, or sophisticated equipment. Thus, we describe a simple, inexpensive, and time-efficient method that may have broader applications for use with other fastidious bacteria.

## OBSERVATION

The genetic modification of bacteria is widely used to assist our understanding of their physiology and pathogenesis. For many years, the absence of a method to introduce foreign DNA and inactivate genes in the gastric pathogen *Helicobacter pylori* represented a major roadblock in our understanding of this bacterium. This was, however, overcome by a technique involving the electro-transformation of bacteria using suicide plasmids, as first conceived by Agnès Labigne (1), then adopted and refined by other workers (2–4). Nevertheless, a limiting factor in the genetic manipulation of *H. pylori* is that a minority of strains is naturally competent for transformation, as shown by an early study, in which only 10% (1/10) of “fresh” clinical isolates were consistently transformable (1), whereas a more recent work found 25% (3/12) of isolates to be transformable (5; unpublished data). Another major issue is that *H. pylori* has fastidious growth requirements and is slow growing, requiring up to 48 h incubation under microaerobic conditions after resuscitation from −80°C. This is generally followed by two or more subcultures, each involving 24–36 h incubation, prior to experimentation. The production of genetically modified bacteria is lengthy, taking at least 5 days for subculture and then plating on selective media, prior to a further 4–5 days to isolate antibiotic-resistant transformants. We, therefore, sought to develop a more rapid, time-efficient, and convenient technique to prepare *H. pylori* bacteria that could be preserved at −80°C and transformed as needed.

To do this, we adapted a protocol used to prepare *H. pylori* bacteria for electroporation (1). In that protocol, *H. pylori* are washed in a glycerol/sucrose solution and stored in super optimal broth with glucose added for catabolite repression (SOC). In the current report, we describe the adaptation of this treatment, allowing a time- and resource-saving method of preparing *H. pylori* bacteria for use in natural transformation experiments. We demonstrate that the method results in bacteria that are competent for at least 6 months at −80°C and is applicable to *H. pylori* strains of varying levels of natural competence.

### *H. pylori* bacteria remain competent after storage at –80°C

**Fig 1:**
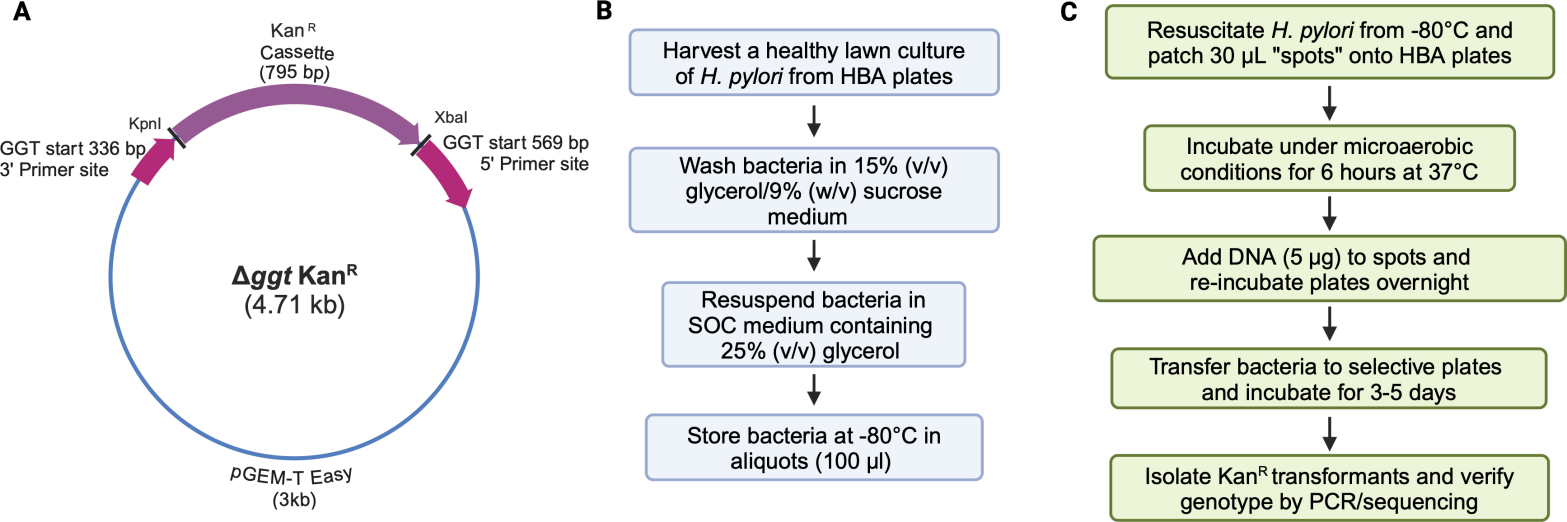
Schematic representation of the protocol used to generate transformants (created with BioRender.com). (**A**) Map of the suicide Δ*ggt* plasmid used in the transformations. Forward and reverse sequences of the PCR/sequencing used to confirm transformants: 5′-ATGAGACGGAGTTTTTTGAAAACG-3′ and 5′-TTAAAATTCTTTCCTTGGATCCG-3′, respectively. (**B**) Competent *H. pylori* bacteria are stored at −80°C until needed. (**C**) Aliquots of thawed bacteria are directly added to HBA plates in “spots” and transformed by the direct addition of DNA.

To test whether competent *H. pylori* could be stored at −80°C and remain capable of undergoing natural transformation, we performed a pilot study. For this, *H. pylori* strain 251 bacteria were harvested from horse blood agar (HBA) plates containing Skirrow’s selective supplement and washed three times in 15% (vol/vol) glycerol/9% (wt/vol) sucrose solution (1). The washed bacteria were resuspended in SOC buffer (2% (wt/vol) tryptone, 0.5% (wt/vol) yeast extract, 10 mM NaCl, 2.5 mM KCl, 10 mM MgCl_2_, 10 mM MgSO_4_, 20 mM glucose) (6), containing 25% (vol/vol) glycerol, and stored at −80°C for 5 days. Transformations were performed using a suicide plasmid (this study), as outlined in Fig. 1. The plasmid comprised a pGEM-T easy backbone into which was cloned two fragments of the *ggt* gene, encoding gamma-glutamyl transpeptidase, and a non-polar kanamycin cassette (Fig. 1A). The pilot study demonstrated that *H. pylori* bacteria stored for 5 days at −80°C remained competent.

### Competent *H. pylori* strains remained transformable over time

Next, we determined whether competent *H. pylori* bacteria could be stored over long periods at −80°C, using five *H*. *pylori* laboratory strains, each with varying levels of natural competence: clinical isolates (251, 10700/PMSS1), as well as reference (26695) and mouse-colonizing (SS1, X47-2AL) strains. Bacteria were prepared as above, and stock preparations were assayed for viability prior to storage at −80°C (Fig. 1B).

**Fig 2:**
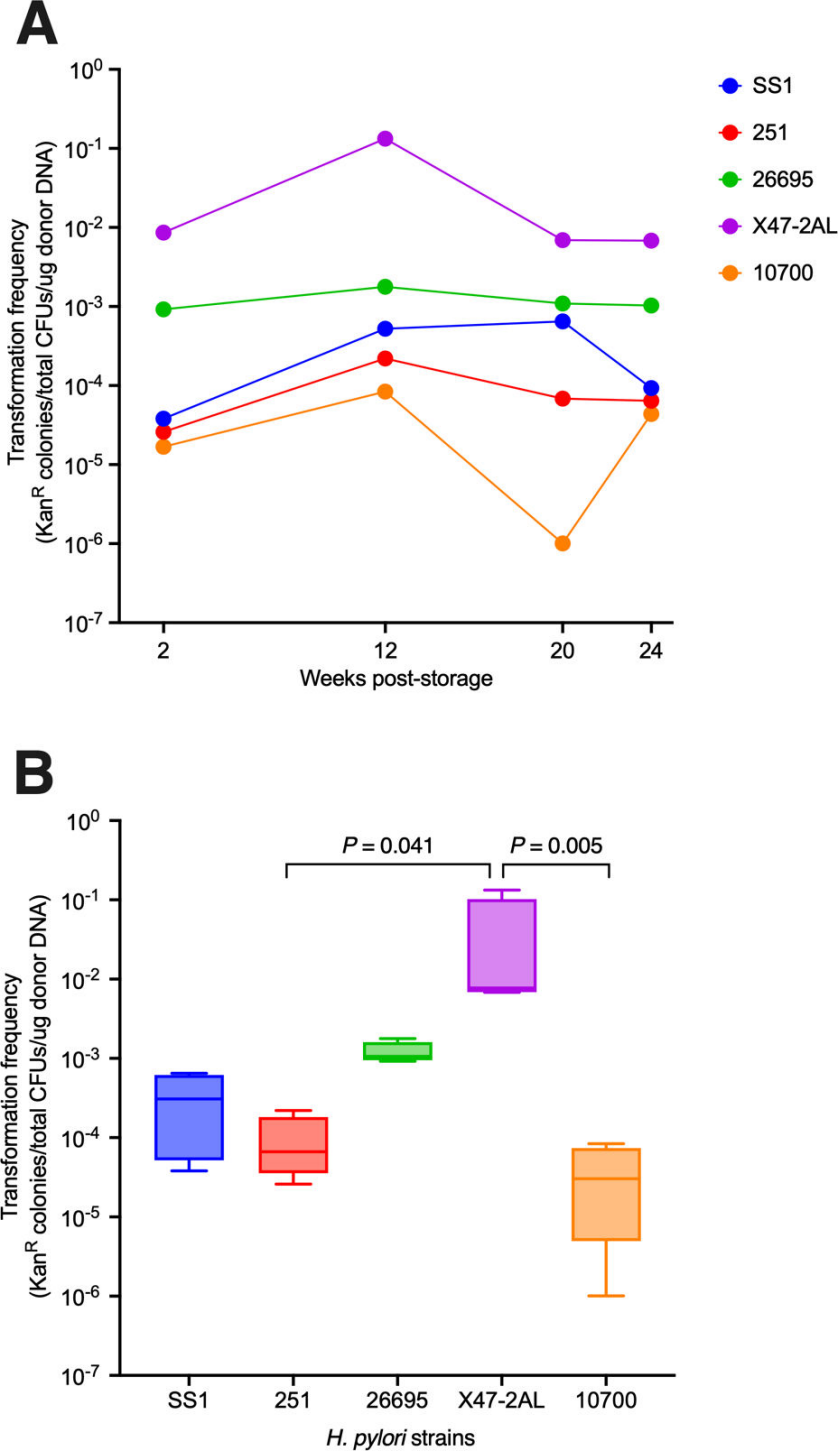
Transformation efficiencies of bacteria prepared from −80°C stocks of various *H. pylori* strains. (**A**) Transformation efficiencies of the *H. pylori* strains at different time points. The points correspond to data from a single transformation, in which the CFUs of kanamycin-resistant (Kan^R^) transformants were determined in duplicate by plate counting (5). (**B**) Box and whiskers plot (with 10%–90% percentiles shown) of the combined transformation efficiencies for each *H. pylori* strain, over time. Plots for each strain were generated by pooling the data from the four time points in panel A. Bacteria were transformed with the Δ*ggt* plasmid (Fig. 1A; 5 µg). Transformation efficiencies were calculated by determining the number of Kan^R^ colonies divided by the total CFUs, per µg DNA. *P*-values were determined by the Kruskal–Wallis test.

To assess whether there were any changes in the transformation efficiency of strains over time, stored bacteria were thawed and a plate transformation procedure (4) was performed at weeks 2, 8, 12, 20, and 24 using the *ggt* suicide plasmid (Fig. 1C). Each strain maintained similar levels of transformation efficiency throughout the 24 weeks (Fig. 2A). Comparisons of the transformation efficiencies for each strain indicated that the laboratory-adapted X47-2AL strain had the highest transformation efficiency of the strains tested (10^−2^–10^−1^ transformants/total colony-forming units (CFUs)/µg donor DNA), whereas clinical isolates 251 and 10700/PMSS1 had the lowest (10^−6^–10^−4^ transformants/total CFUs/µg donor DNA). Taken together, these results suggest that despite differences in the transformation efficiencies of individual strains, all were transformable for up to 6 months storage at −80°C.

We describe here a more rapid and convenient method to genetically manipulate *H. pylori* bacteria. Other studies have focused on improving transformation efficiency by genetic “tricks,” such as by creating genetically modified resistance cassettes that impede the restriction-modification systems that would normally prevent recombination events (7). Such improvements in transformation efficiency are one way to facilitate the genetic manipulation of *H. pylori*; however, these involve complicated molecular biology techniques and do not circumvent the long culture times required to grow the bacterium from frozen stocks.

In the current study, we adapted an established protocol used in the electro-transformation of *H. pylori* (1) to prepare bacteria that maintain their transformation competence over long periods of time. This protocol relies on resuspending bacteria in SOC medium containing bivalent cations (i.e., MgCl_2_), which is known to improve bacterial competence by counteracting the negative charge associated with bacterial membranes, thereby promoting the uptake of donor DNA into bacterial cells (6). It is possible that DNA uptake might be further enhanced using media containing cations commonly employed for the transformation of *E. coli* (e.g., CaCl_2,_ Mn^2+^, and Rb^+^); however, their ability to promote competence in *H. pylori* is not known. The recovery of competent bacteria following transformation was ensured using SOC medium, which has a high nutrient content. Glycerol was added to the resuspension medium as it is known to protect cells during cryopreservation by stabilizing membranes and maintaining stock viability. Further studies of the percentage of glycerol used in the SOC would clarify whether competent *H. pylori* bacteria could be preserved for even longer while maintaining high transformation efficiency.

Interestingly, we observed some increases in the transformation efficiency of competent bacteria between 2 and 12 weeks storage at −80°C (Fig. 2A). Given that environmental stressors are known to increase transformation efficiency in Gram-negative bacteria (6, 8), it is possible that the stress of cryopreservation and thawing on bacterial membranes may have contributed to enhanced uptake of donor DNA by the bacteria and improvement in transformation efficiency. Further experimentation is required to determine whether the differences in transformation efficiency observed between 2 and 12 weeks are statistically significant.

Transformation efficiencies for all *H. pylori* strains tested here were comparable to previous studies, reporting 10^−6^–10^−3^ transformants/total CFUs/µg donor DNA (4, 9). Importantly, our results indicate that competent bacteria stored for up to 6 months post-preparation remain transformable with efficiencies at the upper end of this range (Fig. 2A). This is a great improvement to current methods because it halves the culture time involved for preparing and producing genetically engineered *H. pylori* without compromising the transformation efficiency and without the need for costly materials. It also reduces researchers’ time, as well as the expenses associated with the blood and gas-generating kits needed to grow the bacteria.

As expected, we observed strain-specific variabilities in transformation efficiency, with significantly higher efficiencies observed in the laboratory-adapted *H. pylori* strain X47-2AL, compared with the two clinical isolates, 251 and 10700/PMSS1 (Fig. 2B). Our results are consistent with the genetic diversity of *H. pylori* and reflect the variations in their abilities to exchange molecular material. We report here that many different strains of *H. pylori* can indeed be stored as competent bacteria for at least 6 months and remain capable of natural transformation on the day of thawing. Our approach is simple and time- and cost-effective. We suggest that similar methods may be adapted to other naturally competent and fastidious pathogens requiring ultra-low temperatures for long-term preservation, such as *Campylobacter jejuni* (10), *Streptococcus pneumoniae* (11), *Neisseria* spp., and *Haemophilus* spp. (12).
